# Supplementary material for: The Role of Hydrogen Incorporation into Amorphous Carbon Films in the Change of the Secondary Electron Yield
Source: Int J Mol Sci. 2023 Aug 20;24(16):12999. doi: 10.3390/ijms241612999 (PMC10456004; doi:10.3390/ijms241612999)
Supplement: Supplementary file 1 [file ijms-24-12999-s001.zip › ijms-2537517-supplementary.pdf]

The role of hydrogen contamination on the secondary electron emission properties of amorphous carbon films

N. Bundaleski[1], C.F. Adame[1], E. Alves[2,3], N.P. Barradas[2,3], M.F. Cerqueira[4,5], J. Deuermeier[6], Y. Delaup[9], A.M. Ferraria[7,8], I.M.M. Ferreira[6], H. Neupert[9], M. Himmerlich[9], A.M.B. do Rego[7,8], M. Rimoldi[9], O.M.N.D. Teodoro[1], M. Vasilevskiy[4,5], P. Costa Pinto[9]*

[1]CEFITEC, Departamento de Física, Faculdade de Ciências e Tecnologia, Universidade Nova de Lisboa, Campus de Caparica, 2829-516 Caparica, Portugal
[2]DECN, Instituto Superior Técnico, University of Lisbon, Bobadela 2695-066, Portugal
[3]IPFN, Instituto Superior Técnico, University of Lisbon, Lisbon 1049-001, Portugal
[4]Centre of Physics of the Universities of Minho and Porto (CF-UM-UP) & Laboratorio de Física para Materiais e Tecnologias Emergentes (LaPMET), Braga, 4710-057, Portugal
[5]International Iberian Nanotechnology Laboratory (INL), Av. Mestre José Veiga, 4715-330 Braga, Portugal
[6]i3N/CENIMAT, Departamento de Ciência dos Materiais, Faculdade de Ciências e Tecnologia, Universidade Nova de Lisboa, Campus de Caparica, 2829-516 Caparica, Portugal
[7]iBB—Institute for Bioengineering and Biosciences and Departamento de Engenharia Química, Instituto Superior Técnico, Universidade de Lisboa, Av. Rovisco Pais, 1049-001 Lisbon, Portugal
[8]Associate Laboratory i4HB—Institute for Health and Bioeconomy at Instituto Superior Técnico, Universidade de Lisboa, Av. Rovisco Pais, 1049-001 Lisbon, Portugal.
[9] European Organization for Nuclear Research, CERN, 1211 Geneva 23, Switzerland

[*]Corresponding author: Tel: +41 754 114 468; e-mail: Pedro.Costa.Pinto@cern.ch

Supplementary information

S1. Raman measurements

S1.1 Fittings of the Raman spectra taken with 532 nm laser from all samples apart from 10D

The Raman spectra were fitted, after the background subtraction, by a home-written software. A set of Lorentzian functions (with the peak positions, amplitudes and FWHMs treated as fitting parameters) was used to describe the experimental spectra. For each Lorentzian function, the choice of optimised fitting parameters was made by minimizing the difference between the experimental Raman spectrum and the fit. The results of the fittings are summarized in Table S1.

Table S1. The results of the fitting of the Raman spectra taken from samples deposited on Si using the 532 nm laser with the power of 0.7 W.

Sample	G band position	D band position	FWHM G	FWHM D	I_D/I_G
**reference**	1563 cm^{-1}	1358 cm^{-1}	250 cm^{-1}	300 cm^{-1}	0.73
**0.2D**	1555 cm^{-1}	1360 cm^{-1}	200 cm^{-1}	300 cm^{-1}	0.63
**0.5D**	1545 cm^{-1}	1360 cm^{-1}	180 cm^{-1}	300 cm^{-1}	0.46
**1D**	1540 cm^{-1}	1360 cm^{-1}	200 cm^{-1}	300 cm^{-1}	0.44

S1.2 Raman spectroscopy of the 10D sample taken with 532 nm laser

A Raman spectrum taken from the sample 10 D deposited on silicon is presented in Fig. S1. It is characterised by an intense wide hump attributed to photoluminescence signal typical for hydrogenated amorphous carbon [1], which completely overlaps the region in which G and D bands are expected. A sharp peak at about 520 cm^{-1} is readily attributed to the silicon substrate. The background of a polynomial form was removed using the data analysis and acquisition software WITec Control FIVE ver. 5.1, which introduces considerable uncertainty into the fitting process. The result presented in Fig. S2 reveals an asymmetric structure that can be fitted to two

contributions: the G band at 1550 cm^{-1}, and another peak at about 2100 cm^{-1}, which could be attributed to the C-D$_x$ stretching band. The most striking feature is apparent disappearance of the D band in the spectrum.

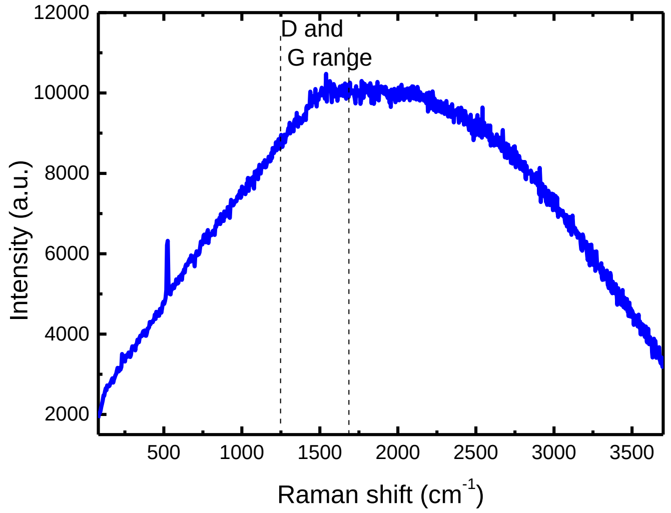

Figure S1. Raman spectrum taken from the sample 10D deposited on Si.

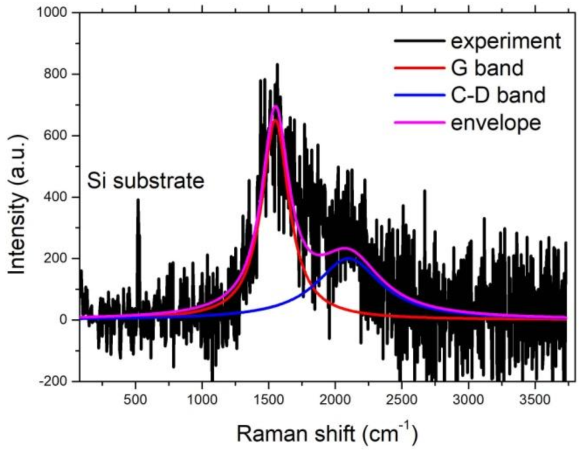

Figure S2. Raman spectrum of the 10D sample obtained after removal of the polynomial background.

S1.3 Raman measurements with different wavelengths

The Raman spectra taken from the same spot of the sample 0.5D using lasers with 633 nm and 532 nm are presented in Fig. S3. Clearly, the wide hump at 2800 cm^{-1} is clearly pronounced only in the spectrum obtained using the 532 nm laser. Therefore, we conclude that this structure is a photoluminescence signal. At the same time the signal at about 2000 cm^{-1} is present in both spectra, indicating that at least part of that structure should be attributed to the C-D$_x$ stretching band [2].

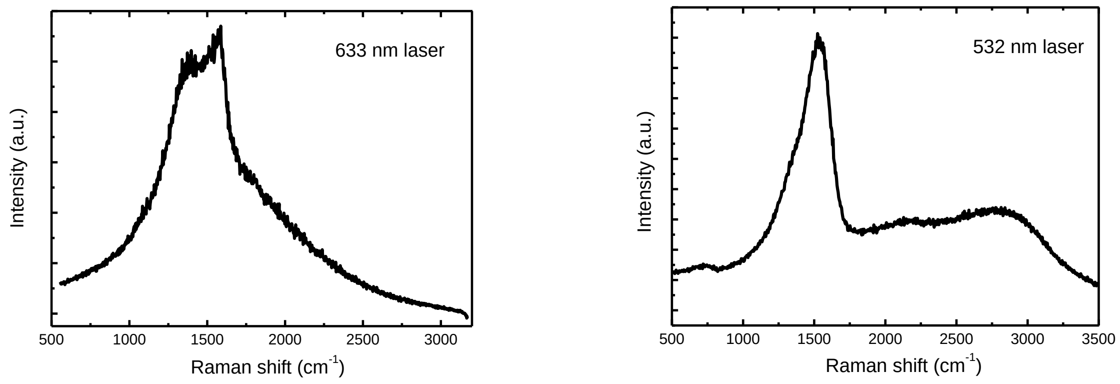

Figure S3. Comparison of Raman spectra taken from the sample 0.5D using two lasers with different wavelengths.

S2. FTIR measurements

S2.1 Experimental results

FTIR measurements of the carbon coatings were taken in the conventional reflection and transmission modes, as well as in the attenuated total reflection mode with a Ge crystal. Measurements of the coatings deposited on quartz were strongly affected by the signal from the substrate. The best results were obtained from the samples deposited on Si substrate in the conventional transmission mode. These results are presented in Fig. S4 revealing clear features that can be attributed to C-D stretching vibrations around 2100 cm^{-1}, only in the case of the sample 10D.

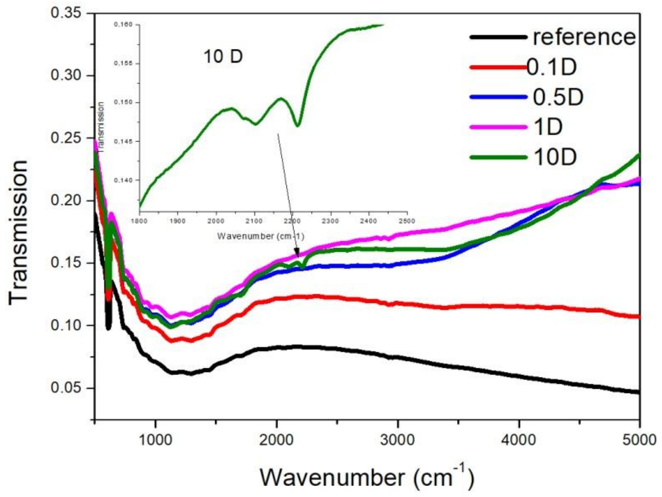

Figure S4. FTIR spectra taken from carbon coatings deposited on silicon.

The 1 – T(ω) spectrum taken from the sample 10D deposited on silicon is presented in Fig. S5. The observed feature was fitted to two Lorentzians after removing the background. The fitting results are shown in Table S2. The two dominant lines are situated at 2214 cm^{-1} and 2109 cm^{-1}, being bellow the two features in HREELS spectra attributed to C-D bonds for about 50 cm^{-1} (i.e. 6.2 meV). The two dominant lines L1 and L2 in the FTIR spectra can be attributed correspondingly to $C(sp^2)$-D and $C(sp^3)$-D bonds, respectively [2]. It appears that about 54 % of the deuterium detected by FTIR is bonded to sp^2 carbon. This value is similar to the one obtained for the 1D sample using HREELS (64 %).

Table S2. Fitting of the FTIR spectrum taken in conventional transmission regime from the sample 10D deposited on silicon.

Component	Frequency (cm^{-1})	FWHM (cm^{-1})	Area $(cm^{-1})^2$
L1	2214	53	0.620
L2	2107	88	0.534

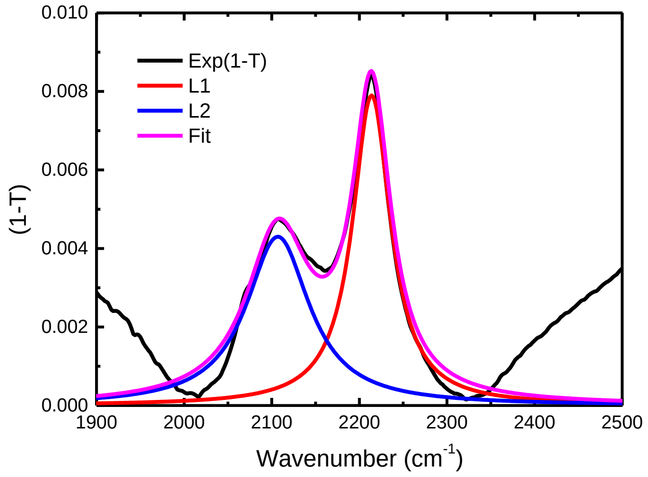

Figure S5. Fitting of the FTIR spectrum taken from the sample 10D in the range expected for the C-D bonds

S2. 2 IR activity of D-C vs. H-C bonds

Infrared (IR) response of materials containing heteropolar covalent bonds like C-H is determined by atomic vibration modes corresponding to their stretching/compression (so called stretching modes). Considering such a bond as a damped harmonic oscillator, the bond polarisability is given by:

$$\alpha_0(\omega) = \frac{q^2}{\mu(\omega_0{}^2 - \omega^2 - i\omega\gamma)} \quad (1)$$

where ω_0 is the stretching mode frequency, γ is a damping parameter, q is the effective charge of the H/D atom, and $\mu = mM/(m + M)$ is the effective mass of the atomic pair (with m and M being the masses of the hydrogen/deuterium and carbon atoms, respectively).

The IR response of a non-polar material containing some polarisable entities can be described by an effective dielectric function, $\varepsilon^*(\omega)$, given by the Clausius - Mossotti formula:

$$\frac{\varepsilon^*(\omega) - \varepsilon_h}{\varepsilon^*(\omega) + 2\varepsilon_h} = \frac{4\pi}{3} \cdot N\alpha_0(\omega) \quad (2)$$

where ε_h is the host dielectric constant and N is the concentration of the heteropolar bonds (i.e, those of H or D atoms). The IR absorption coefficient of a C:H or C:D film can be calculated as

$$\alpha(\omega) = \frac{\sqrt{\varepsilon_h}\,\omega Im\{\varepsilon^*(\omega)\}}{c} \quad (3)$$

Using the expressions (1-3) the absorption coefficient at the resonant frequency becomes

$$\alpha(\omega_0) \approx \frac{4\pi\sqrt{\varepsilon_h}\,q^2 N}{c\mu\gamma}, \quad (4)$$

after assuming low concentration of the polar bonds i.e. $(\omega_0 \cdot \gamma \cdot \mu)^2 >> (4\pi N q^2/3)^2$. We notice that $\alpha(\omega_0) \sim \mu^{-1}$ and $\mu_D/\mu_H \approx 1.9$. Moreover, the damping parameter γ should be larger for deuterium

because its vibration frequency is smaller by a factor of $(\mu_D/\mu_H)^{1/2}$. In addition, one should expect more channels for an anharmonic decay when a vibrating atom is inserted into a solid composed of heavier atoms. Therefore, one should expect that the absorption coefficient due to stretching vibrations of deuterium, at peak frequency, to be 3–4 times smaller than the corresponding value for C:H films, a consequence of the isotope effect.

References

[1] Rusli and J. Robertson, G.A.J. Amaratunga, Photoluminescence behavior of hydrogenated amorphous carbon, J. Appl. Phys. 80 (1996) 2998-3003

[2] M. Rei Vilar, A.M. Botelho do Rego, J. Lopes da Silva, F. Abel, V. Quillet, M. Schott, S. Petitjean, R. Jérôme, Quantitative Analysis of Polymer Surfaces and Films Using Elastic Recoil Detection Analysis (ERDA), Fourier Transform Infrared Spectroscopy (FTIRS), and High-Resolution Electron Energy Loss Spectroscopy (HREELS) Macromolecules 27 (1994) 5900-5906
